# An Injectable, Self-Healing, Adhesive Multifunctional Hydrogel Promotes Bacteria-Infected Wound Healing

**DOI:** 10.3390/polym16101316

**Published:** 2024-05-08

**Authors:** Ling Zhang, Yan Wang, Mingrui Yang, Wen Yu, Zheng Zhao, Yichao Liu

**Affiliations:** 1 State Key Laboratory of Advanced Technology for Materials Synthesis and Processing, Wuhan University of Technology, Wuhan 430070, Chinayanwang2021@whut.edu.cn (Y.W.);; 2 Hospital of Wuhan University of Technology, Wuhan 430070, China; 3 Hainan Institute, Wuhan University of Technology, Sanya 572000, China; 4 Center for Evidence-Based and Translational Medicine, Zhongnan Hospital of Wuhan University, Wuhan 430070, China

**Keywords:** infected wound, self-healing, adhesive, antibacterial, wound healing

## Abstract

Bacterial infections have a serious impact on public health. It is urgent to develop antibacterial hydrogels with good biocompatibility to reduce the use of antibiotics. In this study, poly(lipoic acid-co-sodium lipoate)–phytic acid (P(LA-SL)-PA) hydrogels are prepared by a simple mixture of the natural small molecules lipoic acid (LA) and phytic acid (PA) in a mild and green reaction environment. The crosslinking network is constructed through the connection of covalent disulfide bonds as well as the hydrogen bonds, which endow the injectable and self-healing properties. The P(LA-SL)-PA hydrogels exhibit an adjustable compression modulus and adhesion. The in vitro agar plates assay indicates that the antibacterial rate of hydrogels against *Escherichia coli* and *Staphylococcus aureus* is close to 95%. In the rat-infected wound model, the P(LA-SL)-PA hydrogels adhere closely to the tissue and promote epithelialization and collagen deposition with a significant effect on wound healing. These results prove that the P(LA-SL)-PA hydrogels could act as effective wound dressings for promoting the healing of infected wounds.

## 1. Introduction

Skin tissue is the body’s first defense barrier, which can prevent the invasion of pathogens such as bacteria, viruses and parasites [1,2,3]. The injury of skin tissue easily leads to bacterial infection, resulting in wound bleeding, suppuration, necrosis and even sepsis, which seriously endanger human health [4,5,6]. Recently, antimicrobial hydrogels have played an important role in the management of infected wounds [7,8], maintaining a physiologically moist environment attributed to their high hydration [9], absorbing wound exudate and providing a reliable condition for wound healing with a great application potential in the management of infected wounds [10,11,12,13].

At present, the abuse of antibiotics is resulting in the presence of drug-resistant bacteria [14], which greatly weakens the antibacterial effect, increases cytotoxicity and aggravates wound infection [15,16]. Using raw materials with inherent antibacterial properties has become an effective way to prepare inherently antibacterial hydrogels, which can not only effectively prevent bacteria from developing drug resistance but also have good cytocompatibility on account of their simple composition [17,18]. Inherently antibacterial materials like metal ions or other cationic groups can achieve antibacterial effects with complex chemical modification and potential toxicity inevitably [19,20,21,22]. Indeed, insufficient adhesion and complex synthesis methods make it difficult to adapt to the complex environment of wounds [19,23,24]. Therefore, an inherently antibacterial hydrogel developed using a green and simple synthesis method with excellent antibacterial properties, biocompatibility and multifunctional properties has good potential applications [25,26,27].

Lipoic acid (LA), a natural small molecule, is easy to obtain and directly polymerizes to form hydrogels without toxic crosslinking agents and is widely used in antibacterial, antioxidant and anticancer applications [28,29,30,31]. The poly(lipoic acid-co-sodium lipoate) (P(LA-SL)) hydrogel was obtained by ring-opening polymerization of LA monomers at melting temperature, followed by hydrogen bond formation [28]. However, the P(LA-SL) hydrogel undergoes a sol–gel transition, lacking considerable mechanical strength and stability [32]. In addition, the deficiency of electrical conductivity and adhesion limits the clinical application of these hydrogels in wound healing [23,33].

Phytic acid (PA) is a biomass-derived, environmentally friendly and non-toxic material, which is widely found in beans and seeds [34,35,36]. In addition, a large number of free hydrogen ions released from PA can give the hydrogel wound dressing good electrical conductivity [37], promoting the transmission of electrical signals in the tissue around the wound [38]. Ample hydrogen bond donors and acceptor sites are bonded with water through hydrogen bonds [39]. Abundant phosphate groups of PA can interact with bacterial proteins and chelate with metal ions such as Ca^2+^ and Mg^2+^ to alter the permeability of bacterial cell walls [40]. Therefore, the combination of PA with the P(LA-SL) hydrogel can improve its antibacterial properties and conductivity.

**Figure 1:**
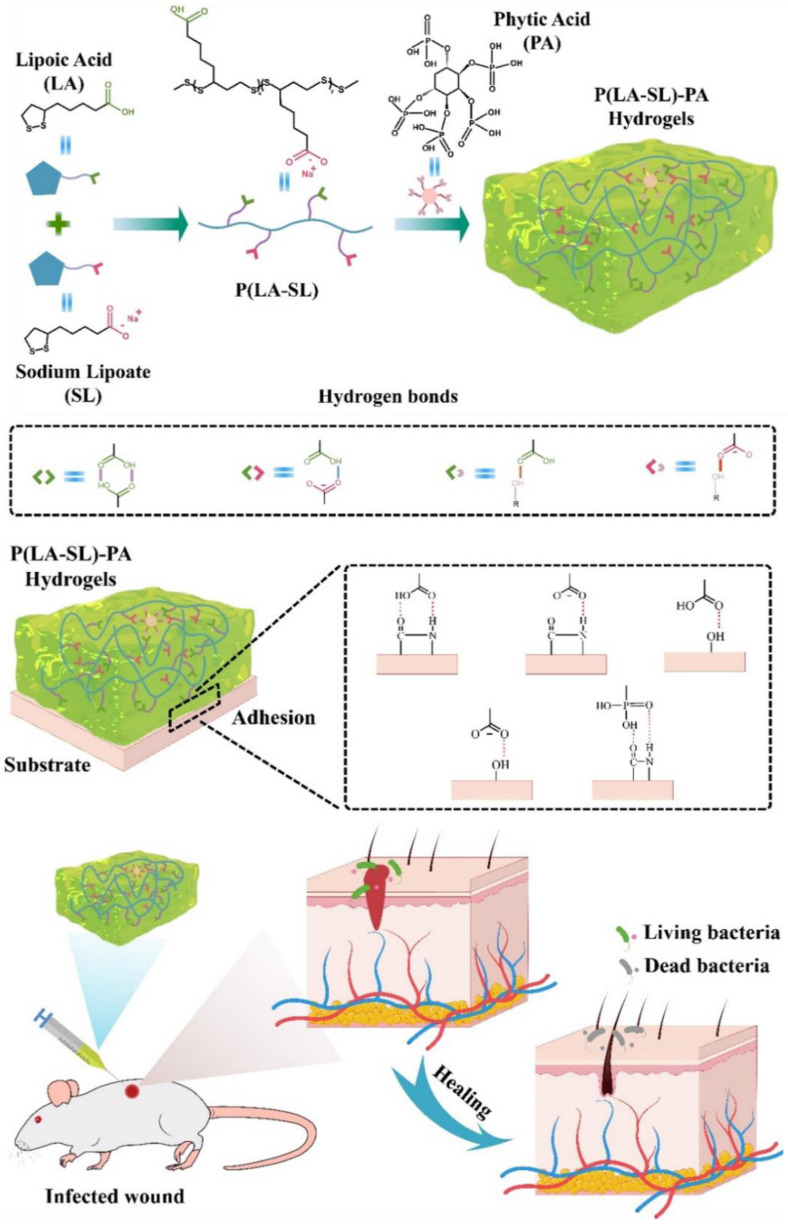
Schematic diagram of preparation process of the P(LA-SL)-PA hydrogel and its application for promoting wound healing of a full-layer skin defect model infected with *Staphylococcus aureus*.

In this study, we adopted a simple green method to prepare antibacterial P(LA-SL)-PA hydrogels that can be locally injected, self-healing and tissue-adhesive. Firstly, LA is dissolved in sodium bicarbonate alkaline solution, and ring-opening polymerization occurs at melting temperature. Then, PA is introduced through hydrogen bond interactions to produce the P(LA-SL)-PA hydrogels (Figure 1). The abundant phosphate groups in PA promote the dangling carboxyl groups and -COO^-^ to form more hydrogen bonds [41], providing a more stable three-dimensional porous network structure of hydrogels. The addition of PA not only improves the compression modulus and adhesion properties of the hydrogels but also provides conductivity. The P(LA-SL)-PA hydrogels can be injected locally on the skin surface and firmly adhere to the tissue. Furthermore, the physical and chemical properties and biocompatibility of the P(LA-SL)-PA hydrogels were systematically investigated. Finally, the effects of the P(LA-SL)-PA hydrogels on the wound healing of a full-layer skin defect model infected with *Staphylococcus* aureus in mice were evaluated.

## 2. Materials and Methods

### 2.1. Materials

Lipoic acid (LA), phytic acid (PA) and sodium bicarbonate (NaHCO_3_) were purchased from Macklin. RPMI 1640 medium, fetal bovine serum (FBS), penicillin–streptomycin (PS), Calcein AM staining, Cell counting Kit-8 (CCK-8) and phosphate buffer saline (PBS) were provided by Beyotime Biotechnology (Shanghai, China).

### 2.2. Preparation of P(LA-SL) Hydrogel

A total of 0.928 g of LA was dissolved in sodium bicarbonate solution (4.5 mL) with a concentration of 0.5 M. Then, the P(LA-SL) hydrogel was obtained in 3 h by vigorous stirring at 70 °C.

### 2.3. Preparation of P(LA-SL)-PA Hydrogels

A total of 0.928 g of LA was dissolved in sodium bicarbonate solution (4.5 mL) with a concentration of 0.5 M, which was strongly stirred with a magnetic stirrer to obtain a uniform monomer solution at the melting temperature of 70 °C for 30 min. Then, PA (5, 15, 25 mg) was mixed with LA and NaHCO_3_ (0.50 M) in 4.5 mL of water at 70 °C for 150 min and cooled to room temperature to obtain the targeted P(LA-SL)-PA-1, P(LA-SL)-PA-2 and P(LA-SL)-PA-3 hydrogels.

### 2.4. Characterization of Hydrogels

The spectra of LA, PA powder and P(LA-SL) and P(LA-SL)-PA-3 hydrogels were recorded using Fourier transform infrared spectroscopy (FT-IR, Nexus, Thermo Fisher Scientific, Shanghai, China) over the range of 400–4000 cm^−1^. The morphology of the samples was characterized using a scanning electron microscope (SEM, JSM-IT200, Jeol, Beijing, China).

### 2.5. Mechanical Characterization of Hydrogels

P(LA-SL), P(LA-SL)-PA-1, P(LA-SL)-PA-2 and P(LA-SL)-PA-3 hydrogels were placed in a mold of the same shape (diameter = 16.0 mm, height = 12.0 mm). The compression property was tested by a universal testing machine (Instron MTS, Norwood, MA, USA) at a compression rate of 5 mm/s at room temperature. Every 3 parallel samples were divided into a group, and the calculated results were averaged.

### 2.6. Rheological Test

The rheological properties of P(LA-SL) and P(LA-SL)-PA hydrogels were evaluated at 25 °C using a rotating rheometer (Kinexus Pro+, Malvern Panalytical, UK), and four different tests were performed, including oscillatory frequency scanning, strain amplitude scanning, temperature sweep and alternate step strain scanning [42].

### 2.7. Self-Healing Characterization of Hydrogels

The rheological recovery and macroscopic self-healing tests were selected to assess the self-healing ability of the P(LA-SL)-PA-3 hydrogel. To investigate the self-healing ability of the samples clearly, the hydrogels were stained in red using methyl orange, which has no influence on self-healing. All the hydrogels were cut into half pieces, and then one red half piece and another half piece without dye were joined together end to end to form a whole one.

### 2.8. Injectable Performance of Hydrogels

To evaluate the injectable properties of hydrogels, qualitative analysis of syringe writing and quantitative analysis of rheological oscillatory frequency scanning were performed. To observe the underwater injection of the hydrogel, we injected P(LA-SL)-PA-3 hydrogel into a wet pig skin in a glass bottle filled with water.

### 2.9. Adhesiveness of Hydrogels

The adhesive ability of hydrogels was qualitatively judged by taking the heart, liver, spleen, lung and kidney as the given materials. The adhesive strength of the hydrogels to the slide was quantitatively tested by the shear lap experiment. Moreover, the P(LA-SL)-PA-3 hydrogel was injected into the pig skin to determine its adhesion and underwater adhesion.

### 2.10. Hemolysis Test

The blood of SD rats was extracted. First, the obtained fresh blood was added to the PBS solution and centrifuged for 10 min at 1000 rpm rotational speed. The supernatant was removed, and the process was repeated three times. Then, the red blood cell suspension was diluted to a 5% concentration solution for use. Next, 0.2 g of hydrogel and 200 mL of PBS or DIW were added to 1 mL of red blood cell suspension and incubated at 37 °C in a shaker for 1 h. Finally, the absorbance of all sample supernatants was measured at 545 nm. Here, PBS was used as a negative control and DIW as a positive control. The following formula is used to calculate the hemolysis rate:

$$hemolysis\;ratio=\frac{A_{s}-A_{0}}{A_{100}-A_{0}}$$

*A_s_*, *A*_0_ and *A*_100_, respectively, indicate the absorbance of the hydrogel group, PBS and DIW supernatants at 545 nm.

### 2.11. Cytotoxicity Assay

The cytocompatibility of hydrogels was analyzed by a cell viability assay and a live cell assay. A 100 μL amount of medium containing L929 cells was added to the 96-well plate with an initial cell density of 5.0 × 10^3^ cells/well. After co-incubation at different periods, the cytotoxicity of the hydrogel extract was tested using a CCK-8 kit for 1 d, 3 d and 5 d. The OD value was read at 450 nm by the enzyme-labeling instrument.

To visualize cell proliferation in vitro, L929 cells cultured with hydrogel extract were stained with the Cal-AM kit and further observed with the inverted fluorescent microscope.

### 2.12. Antibacterial Test

To study the antibacterial activity of hydrogels, we selected Gram-positive Staphylococcus aureus and Gram-negative *Escherichia coli*. All experiments were repeated three times.

### 2.13. In Vivo Wound Healing

All animal procedures followed the National Research Council’s Guide for the Care and Use of Laboratory Animals and were approved by the Animal Care and Use Committee of Wuhan University of Technology. The purchased female Kunming rats (8 weeks) were placed in the animal house for one week to adapt to the environment. The full-layer skin injury model of mice was established by making round full-layer skin wounds with a hole punch and then injecting 20 µL of *S. aureus*. After the *S. aureus*-infected wound model was successfully constructed, different treatments were given to the blank group, Tegaderm biomedical dressing group, P(LA-SL) hydrogel group and P(LA-SL)-PA-3 hydrogel group, respectively. The sample size of each group was 12 mice. The wounds of each group were photographed and measured on the 4th, 8th and 12th day after operation. The wound area was measured using ImageJ software (Windows).

$$Remaining\;wound\;area\left(\%\right)=\frac{wound\;area_{N}}{wound\;area_{0}}$$

### 2.14. Histological Analysis of Wound

On the 4th, 8th and 12th day, wound tissues were collected for H&E and Masson staining to observe the histomorphologic changes in the wound and the regeneration of collagen during healing.

### 2.15. Statistical Analysis

All the experimental data derived from three or more independent experiments are expressed as the mean ± standard deviation. One-way ANOVA analysis was used to estimate all the data (*** *p* < 0.001, ** *p* < 0.01, * *p* < 0.05).

## 3. Results

### 3.1. Preparation and Characterization of Hydrogels

**Figure 2:**
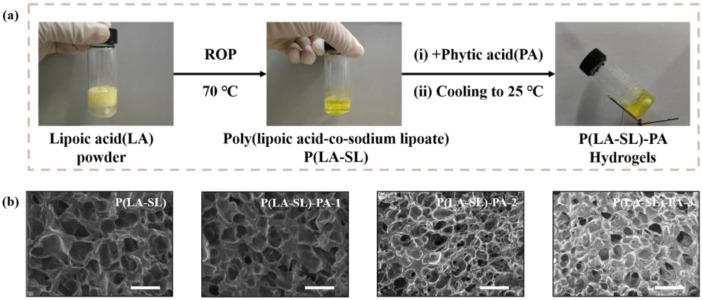
(**a**) The preparation and (**b**) surface morphology observation of the P(LA-SL)-PA hydrogels (scale bar: 50 μm).

In Figure 2, the synthesis route of the poly(lipoic acid-co-sodium lipoate)–phytic acid (P(LA-SL)-PA) hydrogels can be seen by adding PA into the P(LA-SL) solution. In this experiment, the abundant carboxyl groups and -COO^-^ in the side chain of P(LA-SL) effectively crosslinked with the phosphate groups of PA to form various hydrogen bonds to maintain good mechanical properties and structural stability in the P(LA-SL)-PA hydrogels (Figure 1).

**Figure A1:**
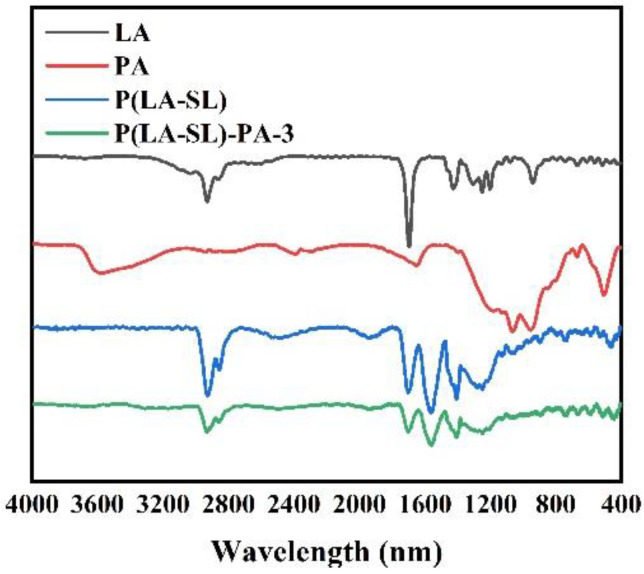
FT-IR spectra of LA, PA, the P(LA-SL) and the P(LA-SL)-PA-3 hydrogels.

In order to investigate the effect of PA on the formation of the P(LA-SL)-PA hydrogels, we prepared four kinds of hydrogels with different contents of PA, including P(LA-SL), P(LA-SL)-PA-1, P(LA-SL)-PA-2 and P(LA-SL)-PA-3 hydrogels, in which the dosage of PA relative to LA was set at 0%, 0.5%, 1.6% and 2.7% (*w*/*w*), respectively. Compared to the P(LA-SL) and P(LA-SL)-PA-3 hydrogels, the C=O peaks of LA powder showed a slight blue shift from 1695 to 1700 and 1702 cm^−1^, and the -OH peaks in the carboxyl group showed a red shift from 1251 to 1247 and 1245 cm^−1^, which may be due to the formation of more hydrogen bonds, as displayed in FTIR. The C=O peaks of the P(LA-SL) and P(LA-SL)-PA-3 hydrogels appeared at 1563 cm^−1^, as a result of the deprotonation of carboxyl groups (Figure A1) [43]. With the introduction of PA, the pore size of the hydrogels decreased, and the structure of the hydrogels became denser and denser, which was related to the degree of crosslinking of the three-dimensional network (Figure 2b). In short, all hydrogels showed a loose porous structure, which was conducive to cell adhesion and migration as well as the transport of nutrients and wastes in the process of skin regeneration.

**Figure 3:**
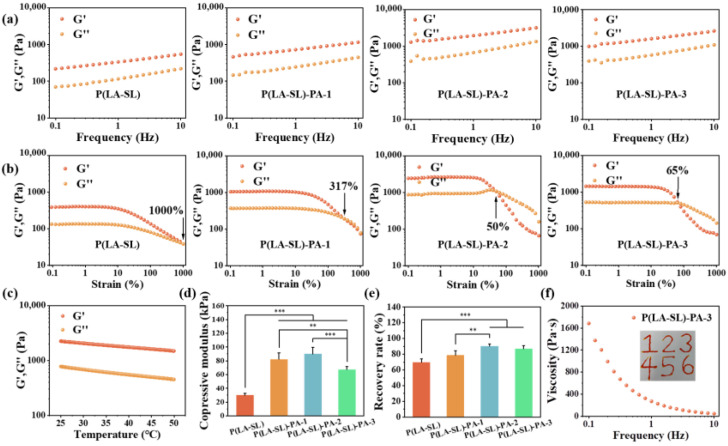
Mechanical properties of the P(LA-SL)-PA hydrogels. (**a**) Frequency sweep measurement; (**b**) strain sweep test; (**c**) temperature sweep test of the P(LA-SL)-PA-3 hydrogel; (**d**) compression modulus; (**e**) deformation recovery rate under 65% strain; (**f**) shear-thinning behavior of the P(LA-SL)-PA-3 hydrogel. n = 6, ** *p* < 0.01, *** *p* < 0.001.

In addition, frequency sweep measurements and strain sweep tests were used to test the dynamic rheological properties. As shown in Figure 3a,b, in the linear elastic region (0.1–10 Hz), the storage modulus G′ and loss modulus G″ of these hydrogels tended to be stable. Moreover, G′ was always greater than G″. These results indicated that these hydrogels formed a stable network structure. The critical point of the P(LA-SL) hydrogel (1000%) was higher than that of the P(LA-SL)-PA-1 hydrogel (317%), the P(LA-SL)-PA-2 hydrogel (50%) and the P(LA-SL)-PA-3 hydrogel (65%) (Figure 3b). The increased crosslinking density caused by the enhanced hydrogen bonding between the P(LA-SL) side chain carboxyl group and PA improved the mechanical properties of the P(LA-SL) hydrogels. The temperature sweep test demonstrated that the P(LA-SL)-PA-3 hydrogel also had stable G′ and G″ values in the temperature range of 25–50 °C (Figure 3c), forming a stable hydrogel.

**Figure 4:**
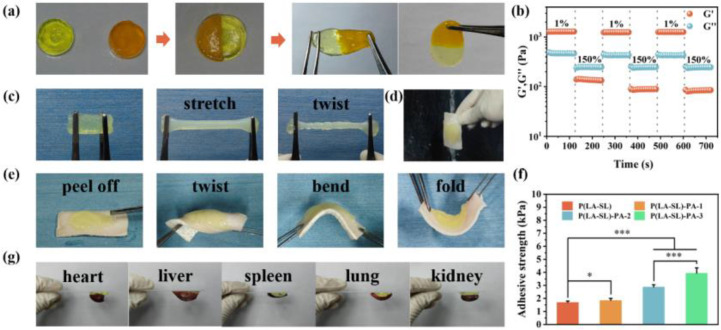
Functionalities of the P(LA-SL)-PA hydrogels. (**a**) Self-healing behavior of the P(LA-SL)-PA-3 hydrogel; (**b**) alternate step strain sweep test of the P(LA-SL)-PA-3 hydrogel between small strain (1%) and large strain (150%) with 90 s for each strain interval; (**c**,**d**) the toughness and wet adhesive property of the P(LA-SL)-PA-3 hydrogel; (**e**) adhesion on the skin under dynamic humid conditions of the P(LA-SL)-PA-3 hydrogel; (**f**) adhesion strength of the P(LA-SL)-PA hydrogels with glass slide; (**g**) tissue adhesion. n = 6, * *p* < 0.05, *** *p* < 0.001.

Proper mechanical properties enable hydrogels to avoid unnecessary damage and better adapt to clinical applications. As shown in Figure 3d, the compression modulus of the P(LA-SL)-PA hydrogels augmented the enhancement of hydrogen bond interaction with the increase in PA content. However, when the PA content was 2.7%, the compression modulus of the hydrogels decreased. This may be because the acidity of PA destroyed the alkaline environment of LA polymerization, and thus, the crosslinking density and the strength of the hydrogels decreased. Hydrogels could withstand compression and bending and recover a certain deformation after 65% compression strain. The P(LA-SL)-PA-3 hydrogel exhibited good toughness and shape stability, exhibiting only slight plastic deformation (87.00 ± 3.99%) (Figure 3e). Moreover, it can be restored to its original state after stretching and twisting (Figure 4c).

### 3.2. Injectability, Self-Healing Ability, Adhesion and Conductivity

**Figure A2:**
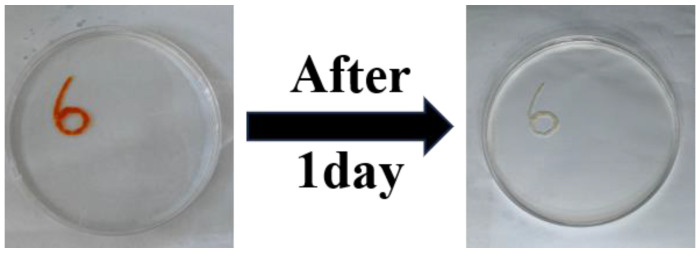
Adhesion of the P(LA-SL)-PA-3 hydrogel 1 day after injection.

The injectable hydrogels facilitate practical biomedical applications, such as easier and more convenient manipulation on wound surfaces [44]. The injectable performance of the hydrogels was tested by qualitative extrusion tests and quantitative shear rheological tests. The results of the rheological tests showed that the viscosity of the P(LA-SL)-PA-3 hydrogel decreased from 1685 to 44.6 Pa·s when the shear frequency increased from 0.1 to 10 Hz. Moreover, it was found that the P(LA-SL)-PA-3 hydrogel with good injectability could be continuously injected into six numbers through a 10 mL syringe with a 0.7 mm needle without blocking, written as the number “123456” (Figure 3f). Notably, the P(LA-SL)-PA-3 hydrogels could be injected into wet pig skin tissue, maintaining good adhesion and remaining hard to peel off under water (Video S1). They were injected into a Petri dish soaked in water and still adhered firmly to the Petri dish for 1 day (Figure A2). Under the action of high shear force, the sol–gel transformation behavior of these hydrogels makes it easier to inject them on wound surfaces and makes them a suitable candidate for wound dressings.

Dynamic disulfide bonds and reversible hydrogen bonds in the network structure endow hydrogels with good self-healing ability. First, the self-healing property of the P(LA-SL)-PA-3 hydrogel was observed through macroscopic observation. As shown in Figure 4a, freshly cut hydrogels were placed together, one of which was stained with methyl orange, and healed by physical contact without external force. The P(LA-SL)-PA-3 hydrogel interface was not clear. The healed hydrogel was able to withstand its own gravity without splitting and still does not separate under large strain. The rheological result is shown in Figure 4b. Under the small strain of 1%, the energy storage modulus G′ of the P(LA-SL)-PA-3 hydrogel (1277 Pa) was greater than the loss modulus G″ (488 Pa). As the strain increased to 150%, G″ (247 Pa) was greater than G′ (143 Pa). These results proved the structural collapse of the P(LA-SL)-PA-3 hydrogel. When the strain reduced again to 1%, G′ rapidly returned to its initial value. The modulus changed stably and alternately in three cycles. These results demonstrated that the P(LA-SL)-PA-3 hydrogel had fast and effective self-healing properties, which could adapt to the tissue during wound repair.

**Figure A3:**
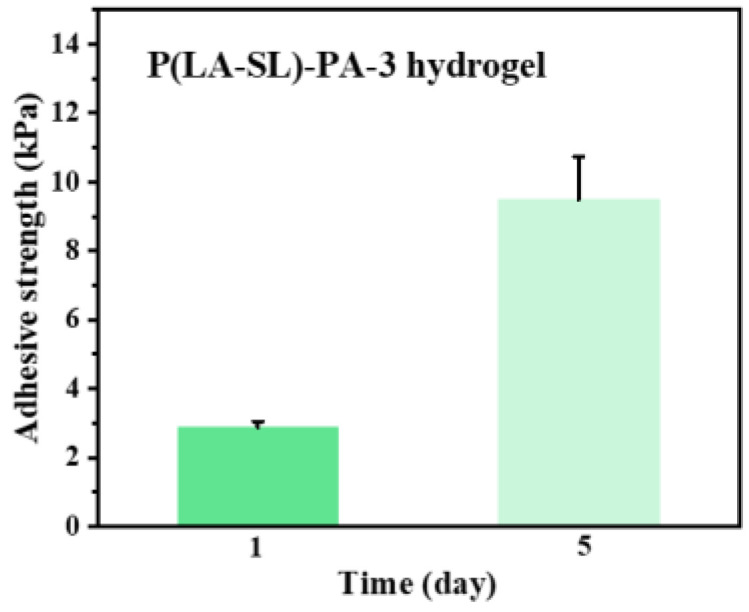
Change in adhesion strength of the P(LA-SL)-PA-3 hydrogel over time.

Adhesive wound dressings can better fit the wound. Due to the abundant -COOH and -OH groups, the P(LA-SL)-PA-3 hydrogel exhibited good adhesion. As shown in Figure 4g, the P(LA-SL)-PA-3 hydrogel adhered firmly to the major organs of mice (heart, liver, spleen, lung and kidney). This adhesion property may be attributed to the formation of Schiff base bonds, electrostatic interaction and hydrogen bonding with skin tissue [41]. The results of the shear lap experiment showed that the adhesion strength increased with the increase in PA content and time (Figure 4f and Figure A3). In addition, PA provides additional hydrogen bond donors that could bind with the tissue and thus enhance the adhesion strength to the skin.

As shown in Figure 4e, the P(LA-SL)-PA-3 hydrogel was injected into dry and wet pig skin tissue. After rotating, folding and bending, the hydrogel injected into dried pig skin tissue still adhered to the pig skin tissue and was hard to peel off. Furthermore, it was injected into the wet pig skin tissue underwater and adhered to the pig skin well. It adhered to the surface of the pig skin firmly even under the impulse of water (Figure 4d). These results indicated the P(LA-SL)-PA-3 hydrogel possessed good underwater injection and adhesion properties (Video S2).

**Figure A4:**
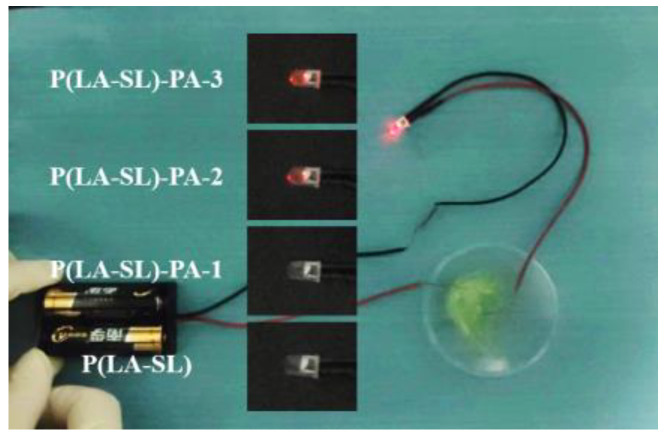
Conductivity of hydrogels and test apparatus.

In addition, PA can provide free hydrogen ions to the P(LA-SL)-PA hydrogels’ network and improve their conductive properties. As shown in Figure A4, the lamps of the P(LA-SL) and the P(LA-SL)-PA-1 hydrogels were dark, while the lamps of the P(LA-SL)-PA-2 and the P(LA-SL)-PA-3 hydrogels were luminous (Video S3). These results indicated that the P(LA-SL)-PA-2 and the P(LA-SL)-PA-3 hydrogels exhibited conductivity owing to the addition of PA. Furthermore, the brightness of the lamp increased with the increase in PA content. The electrical conductivity of the P(LA-SL)-PA-3 hydrogel promoted the transmission of signals from the surrounding tissue and was conducive to wound healing [38]. Due to its good injectable, self-healing, adhesive and conductive properties, the P(LA-SL)-PA-3 hydrogel attached to the wound well and adapted to the dynamic of the wound as a wound dressing for skin repair.

### 3.3. Hemocompatibility and Cytocompatibility

**Figure 5:**
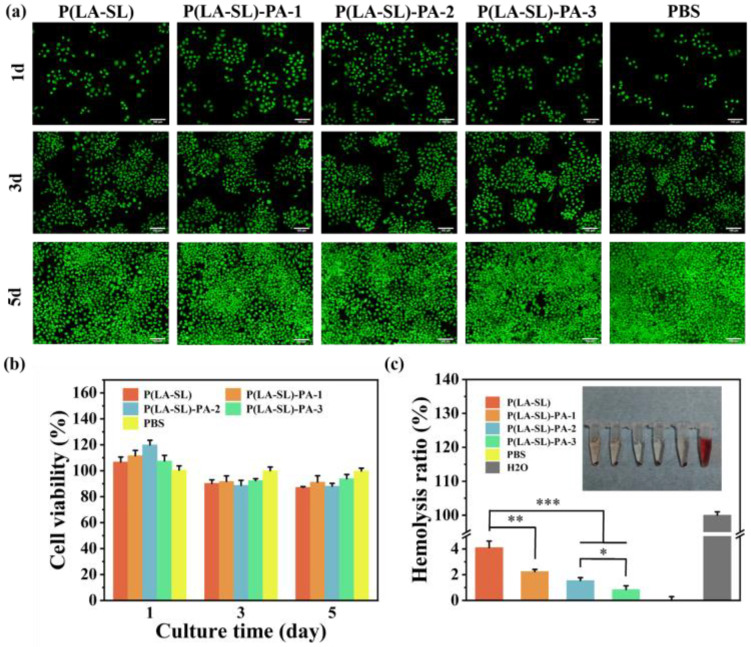
In vitro analysis of biocompatibility. (**a**) The live staining of L929 cells at different times (scale bar = 100 μm); (**b**) the survival rate of L929 cells after 1, 3 and 5 days; (**c**) hemolysis ratio and the inset picture shows the hemolysis assay. n = 4, * *p* < 0.05, ** *p* < 0.01, *** *p* < 0.001.

Good biocompatibility is a prerequisite for the application of these hydrogels in wound repair. In this study, blood compatibility and cytocompatibility were tested to evaluate the biocompatibility of the P(LA-SL)-PA hydrogels. The morphology of L929 cells after 1 day, 3 days and 5 days was qualitatively evaluated by living cell staining (Figure 5a). The number of L929 cells increased significantly. The cells spread out well in the extract and showed a complete spindle structure. Moreover, the cytocompatibility of the hydrogels was evaluated by observing the cell viability of L929 cells. The results showed that the viability of cells cultured with extracts was higher than that of the control group after 1 day of staining with a CCK-8 kit. Even after 3 d and 5 d, the cell viability of the hydrogel groups still remained around 85% (Figure 5b). As shown in Figure 5a, the blood cell morphology remained intact, and the hemolysis rate was less than 5% (Figure 5c). These results suggested that the hydrogels had good blood compatibility. In conclusion, the hydrogels had good biocompatibility and could be applied to wound healing.

### 3.4. Antibacterial Property

**Figure 6:**
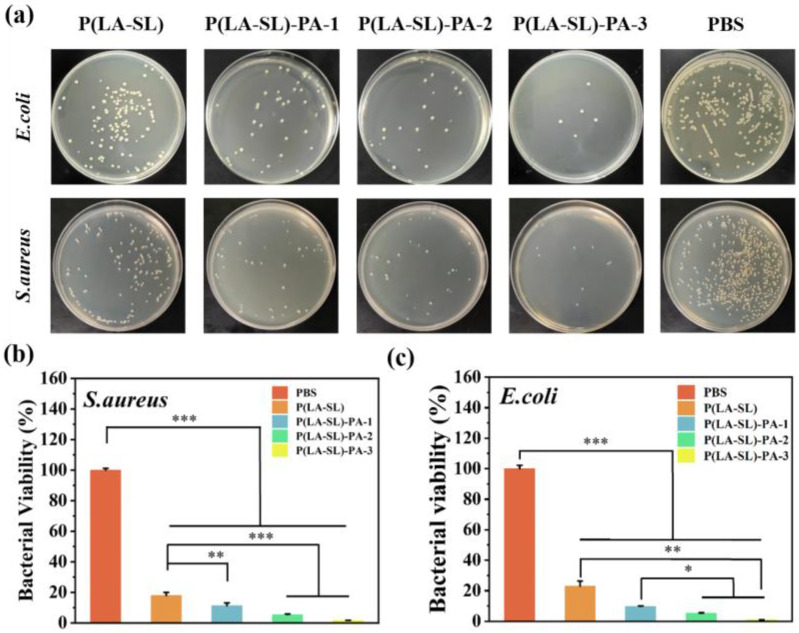
In vitro analysis of antibacterial activity. (**a**) Photographs of the antibacterial effect; (**b**,**c**) the corresponding bacterial survival rate of *E. coli* and *S. aureus*. n = 4, * *p* < 0.05, ** *p* < 0.01, *** *p* < 0.001.

Bacterial infection is a common problem in the process of wound healing and skin regeneration. The inflammatory response caused by bacterial infection will further cause infection-related complications, prolong the wound healing time and reduce the quality of wound healing. Therefore, hydrogels with antibacterial properties are particularly important. We perform colony counts on agar plates. From Figure 6a, the number of colonies in the P(LA-SL) hydrogel group was much less compared with the PBS group, probably because the slightly acidic environment inhibited bacterial growth [23]. As the PA dosage increased, the number of bacterial colonies further decreased. It showed concentration-dependent antibacterial activity. This was due to the abundant phosphate groups of PA, which could not only combine with Ca^2+^ and Mg^2+^ on bacterial membranes to destroy bacterial membrane structure but also chelate with proteins to destroy bacterial organelle function. In addition, H^+^ released by PA has excellent membrane permeability and shows good antibacterial ability by destroying the bacterial membrane. From Figure 6b,c, the survival rate of bacteria in the phytic acid group decreased to 9.72%, 5.10% and 0.95%, which proved that the P(LA-SL)-PA hydrogels had good antibacterial properties, inhibited the growth of bacteria and promoted wound healing.

### 3.5. In Vivo Wound Healing Evaluation and Histological Analysis

**Figure 7:**
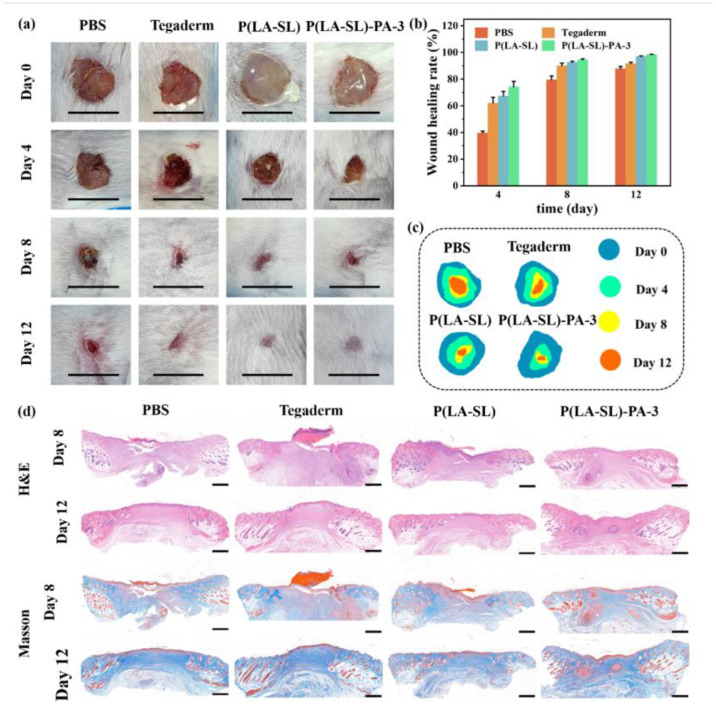
The P(LA-SL)-PA hydrogels enhanced infected wound healing in vivo. (**a**) Photographs of the full-thickness wound on days 0, 4, 8 and 12 for PBS, Tegaderm commercial dressing film, the P(LA-SL) and P(LA-SL)-PA-3 hydrogels (scale bar = 1 cm); (**b**) wound healing rate; (**c**) schematic diagram of the wound area; (**d**) H&E-stained and Masson-stained images of the wound on day 8 and 12 (scale bar = 500 μm).

The proper mechanical properties, good adhesion, biocompatibility and antibacterial properties of hydrogels are more conducive to wound healing. The P(LA-SL)-PA-3 hydrogel was selected for in vivo infected wound evaluation. A full-layer skin defect model in mice was developed to evaluate the therapeutic effect of the P(LA-SL)-PA hydrogels on wound healing. As shown in Figure 7a, it can be clearly seen that the wound area of the P(LA-SL) and the P(LA-SL)-PA-3 hydrogels had a greater degree of contraction than that of the control group, and the P(LA-SL)-PA-3 hydrogel was better than the P(LA-SL) hydrogel. On day 12, the degree of wound shrinkage in the PBS group was much less than that in the P(LA-SL) and P(LA-SL)-PA-3 groups. The wounds of the P(LA-SL) group and the P(LA-SL)-PA-3 group basically healed, and the healing rate of the P(LA-SL)-PA-3 group was higher, reaching 98% (Figure 7b). These results showed that the wound area contraction of hydrogels was faster than that of the PBS and Tegaderm groups, and the healing effect of the hydrogels was significantly better than the other groups. This may be because the hydrogels inhibited the growth of bacteria, accelerated inflammation of the wound and provided electrical conductivity, promoting bacteria-infected wound healing.

The control group and the Tegaderm dressing group still had a large number of inflammatory cells (blue), and there was a significant inflammatory response on day 8 [45,46]. On day 12, it was clearly observed that the P(LA-SL)-P-3 hydrogel-treated group showed an almost complete epidermal structure and a small number of inflammatory cells, as indicated by H&E staining results. The scar width in the Tegaderm dressing group and the control group was larger, and the collagen deposition in the wound tissue was much less than that in the material group. Among them, the collagen fibers in the P(LA-SL)-PA-3 treatment group were the maximum, showing a thin epidermis and new hair follicles on the 12th day (Figure 7d).

In addition, the biocompatible, injectable and self-healing P(LA-SL)-PA-3 hydrogel was more easily injected into the wound site, fit better on the wound surface and adapted to changes in wound tissue. Furthermore, bacterial growth was inhibited, which sped up the end of the inflammatory response and promoted the formation and growth of granulation tissue. The P(LA-SL)-PA-3 hydrogel’s three-dimensional network structure also enabled the transport of nutrients and the elimination of metabolic waste. Its electrical conductivity promoted the transmission of electrical signals from the tissue around the wound [38]. All these factors indicated that the P(LA-SL)-PA-3 hydrogel had a good wound-healing effect, so it had great potential as a dressing for infected wounds.

## 4. Conclusions

In conclusion, we prepared injectable, self-healing P(LA-SL)-PA hydrogels with strong adhesion, conductivity, good antibacterial properties and biocompatibility through a green synthesis method. These properties enabled the P(LA-SL)-PA hydrogels to provide a good repair environment for wounds and accelerate the rapid healing of infected wounds. In addition, in the infected wound model, the P(LA-SL)-PA hydrogels achieved an excellent healing effect, accelerated collagen deposition and epithelialization, inhibited the growth of *S. aureus* and *E. coli* and promoted the healing of infected wounds. Therefore, the P(LA-SL)-PA hydrogels have great potential in diabetic wound treatment and management.

## Data Availability

Data not available due to commercial restrictions.

## Supplementary Materials

The following supporting information can be downloaded at: https://www.mdpi.com/article/10.3390/polym16101316/s1, Video S1: Injection under water; Video S2: Adhesion under water; Video S3: Conductivity.
